# Immanuel Kant's Schema of object perception and cognition

**DOI:** 10.1177/03010066251345679

**Published:** 2025-06-10

**Authors:** Gerald Westheimer

**Affiliations:** University of California, Berkeley, USA

**Keywords:** External objects, mental constructs, representation, brain states

## Abstract

In the *Critique of Pure Reason*, Kant proposed a detailed system of mental processes and constructs that might lead to a person's perceiving and comprehending an object in the outside world. The diffuse and extended original, found largely impenetrable and hence neglected in most modern discourse, is here revisited and presented in an updated contemporary idiom, with the aim of showing some structure in the mental world that may serve as a counterpart to definable states of the real world when attempts are made to find correlations between the two.

## Introduction

When Fechner (1860) conceived psychophysics, he made it quite clear what it was all about. There was a material world and a mental world, and the task was to develop a functional relationship between the two. Fechner saw this taking place in two stages, *outer psychophysics* and *inner psychophysics.* The difference between the two relates to where the corporal world ends and the mental world begins. For outer psychophysics, it is where the stimulus impinges on the body. But Fechner, who had a medical degree, knew that there was an intermediary mediating stage conveying signals into the brain, which is therefore the *immediate* place anchoring the corporal side for the functional relationship with the mental world. In his time, little was known about how stimuli got into the brain and where they went, but Fechner was positive about the existence of this link in his chain, and he expressed confidence of its being elucidated in due course.

Hence in a single paragraph, published in 1860, Fechner laid out the mission of three current disciplines,

**classical psychophysics**, relating stimuli defined in terms of physics to mental states,

**sensory neuroscience**, relating physically defined stimuli to correlated states of neural tissue, and

**cognitive neuroscience**, relating physically defined brain states to mental states.

Since Fechner's time, the physical world has been more and more fully explored, and giant strides have been made in elucidating its representation in the nervous system, particularly in the brain. A functional relationship has, however, two partners. To practice psychophysics or cognitive neuroscience, one has at a minimum to acknowledge the existence of a mental world, although the framing of the inquiry usually does not demand deep knowledge of that world's structure. Often it merely has to act as a simple binary indicator—given a small real-world stimulus perturbation, was a change detected, yes or no? Brindley (1960) was later to call this a Class A experiment.

We can look back on 150 years of success in which cognitive scientists, both in psychology and in neuroscience, used such minimal probing of the mental world while centering their research into brain and behavior on methodologies almost exclusively within the physical realm. But it is becoming increasingly evident that understanding behavior needs guidance by concepts whose origin lies beyond knowledge of the physical world. Gestalt psychology, formulated 100 years ago, serves as an example.

The study of the mind, though etymologically the discipline of psychology, has been the province of philosophy, where the discourse is not a good match to how ideas are argued in the natural sciences. Still, it is essential to plumb the mental world for ideas and concepts that would allow it to be a more equal partner when researching Fechner's functional relationships.

One of the deepest attempts at charting the mental world—virtual, subjective and private as it is—was that of Immanuel Kant, 250 years ago. His main work, *Critique of Pure Reason*, published in two editions, the first (known as *CPR A*) in 1781 and the second (*CPR B*) in 1787, is in part devoted to a taxonomy of processes, steps and stages, replete with carefully defined terms and subtle distinctions, many of direct relevance to cognition of visual objects. Sadly this work is largely inaccessible, for a variety of reasons. The writing in the archaic language of German philosophers of the time is almost impenetrable. Because it has been deemed so important and fundamental, it has been subjected to exegesis second only to the bible, which has discovered that it is not a single unitary work, but a patchwork of passages written as Kant's thinking progressed over a decade or two. All this is made even less transparent by ambiguous and sometimes even misleading terms in the English translations.

Kant's teaching covers a large ground, much of it in areas beyond the scope of this review, but a major part of the *Critique* is a flowchart of the mental processes proposed by Kant of how sensory signals from an object in the real world lead to the recognition of that object by a conscious observer, and of the system of rules pertaining to its inclusion in abstract concepts. For these reasons, the attempt is made here to convey, in a contemporary idiom, in outline the sections of Kant's system that relate to perception and cognition. The aim is to point to those constructs in Kant's mental stream that can then be given sufficient specificity to allow their operational definition, i.e. to establish characterizable and repeatable mental states of alert subjects to enable correlation with physical measurements of brain activity.

Before this can be detailed, the most important fundamentals of Kantian thinking need to be laid out.

### From the Real to the Mental World and its Fundamental Features

Unlike those philosophers, like Bishop Berkeley, who are skeptical or even deny the existence of a world of real objects, or those who like some materialists dismiss mental phenomena, Kant believes in both, albeit as being very distinct. The terms *transcendent* and *transcendental* recur constantly. Organizing processes immanent in the mental world are transcendental. Transcendent, on the other hand, is everything beyond the subject's own mental world such as real objects, i.e. things-by-themselves (*Dinge-an-sich*). Modern scientists have no problem operating in a material world though it is transcendent; the impetus for studying Kant is to examine the extent to which the transcendental can be crisply enough described to lend itself to empirical study.

Another distinction made by Kant is between elements that are pure, sometimes known as or called *a priori*, anything that is inherent in the mind and existing there before any experience, and those whose origin is empirical, also sometimes *a posteriori,* i.e. mental content that arises from or is associated with experience. In particular, *space* and *time* are *a priori* in nature, being brought to mental operations prior to and independent of any experience. This proposition became subject to much debate in the subsequent century but is one on which Kant is very firm.

## Terminology

The primary context of this study is perception and cognition, visual in particular. Although Kant did not limit his discourse to this modality, it seems topmost in much of it. The illustrative examples here reflect this.

Kant uses a very elaborate terminology where a significant number of words are explicitly defined. In the following, they are italicized in their English translation and accompanied on first use by Kant's original. These words in their more common use are avoided elsewhere, to prevent any possible ambiguity. An appended glossary provides citation to their original definition.

Two critical words need comment.

*Anschauung* (literally “looking at”) is usually translated as *intuition*, but this word fails to convey Kant's meaning of belonging to an early receptive stage prior to significant active mental processing. This was understood and commented on by Bertrand Russell (Russell, 1945), whose evaluation of Kant rests more on his wider philosophical pronouncements than on Kant's system of processing incoming sense data, as is the intent here. Kant's most important translator, N. Kemp Smith, while continuing the tradition of translating it as intuition, defines *Anschauung* as “immediate apprehension of a content which, as given, is due to the action of an independently real object upon the mind” (Smith, 1918, p. 104). To avoid the misdirection to which this common translation leads, the word is, therefore, here left in the original German.

*Vorstellung*, (literally “placed in front of”): the usual translation *representation has* the implication of the presentation of something that exists or has previously existed. But Kant uses it more generally (see *B376f* for a clearly articulated hierarchical blueprint). In the current context, it stands for mental constructs that have the capacity of being visualized (“in the mind's eye”). To the extent that they have empirical origin, in particular having been derived from actual external visual targets, they are indeed representations, making the common translation apropos.

Though Kant in the *Critique* is not always clear on this, the word *Object* is employed here for a given entity, from the external target through all constructs embodying its portrayal in the various arenas of the mental world (*“Denn ohne Anschauung fehlt es aller unserer Erkenntnis an Objekten” B87 – “* … without *Anschauung* all of our knowledge would lack objects”).

## The Three Arenas in the Mental World

Broadly speaking, for Kant, the mental world has three hierarchical levels

1. *Sensibility (Sinnlichkeit)* the arena in which *sensory impressions (Sinneseindrucke)* coming in from outside targets are, to use a modern term, preprocessed, generating *Anschauungen.*
2. *Understanding (Verstand)*, the capacity for knowledge, housing *concepts (Begriffe),* that are both empirical and pure, i.e. pre-existing.
3. *Reason (Vernunft)* the arena for higher mental processing, *ideas.*

Because this study is confined to the sensing, perceiving, and (re)cognizing visual objects, only the first of two of these three levels are of concern.

For our purpose, the process begins with an object, outside the observer, signals from which enter the first of the levels as raw *sensory impressions (Sinneseindruecke)*. These are immediately formatted by the *a priori* forms of *time* and *space*, and by a process of *productive imagination (Einbildungskraft)* in which inherent rules from the further stage of *understanding* are brought to bear, to provide order and cohesive form. The result of the synthesis is called *a manifold in the Anschauung (ein Mannigfaltiges in der Anschauung)*, a unified configuration, a whole. Because we are still in the arena of *sensibility* and not yet in that of *understanding,* this *manifold* has not been comprehended as meaning anything in particular.

***Manifolds of Anschauung*** have sufficient structure and unity to be *appearances (Erscheinungen)*, allowing them to be *representations (Vorstellungen)*. It is to be noted that the synthesis generating them utilizes *concepts (Begriffe)*, constructs whose home is the realm of *understandin*g *(Verstand)*, but Kant insists that the *productive imagination* here uses only pure concepts and not those whose origin is empirical.

The ***manifolds*** are now ready to be processed. This is accomplished by a further stage of synthesis, in which *Schemata,* procedures in which innate concepts called *Categories (Kategorien)*, as well as empirical concepts that had been previously laid down in *understanding,* participate in the generation of *perceived objects* (*Wahrnehmungsobjekte* {*Mit Empfindung begleitete Vorstellungen B147*}).

**Figure 1.**
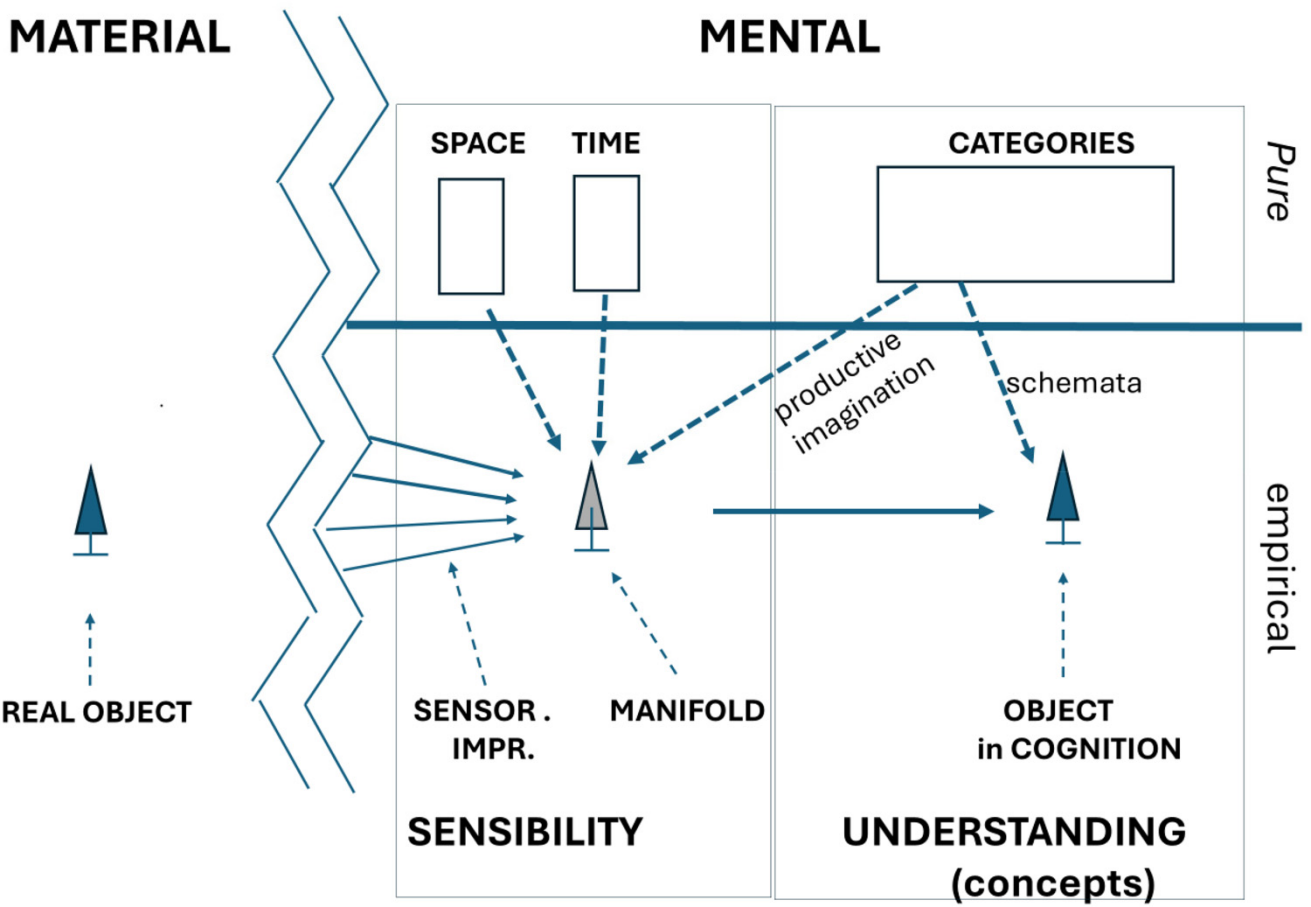
Graphical layout of system of object perception and cognition in Kant's *Critique of pure Reason.* Real world, left; mental world, right; pure or *a priori*, upper; empirical, lower; arena of *sensibility *home of *Anschauungen* and arena of *Understanding *in adjoining boxes; solid arrows indicate direct input, heavy dashed arrows refer to mediating of the pure forms on the empirical.

Figure 1 is an attempt to represent graphically the various levels, processes and constructs, and their interactions.

## Presentation and Representation

Kant is concerned that time enters (as an *a priori* formatting element) to ensure that *manifolds* are unified entities because he felt that consolidation of an object in perception demands some persistence of incoming signals. But, leaving aside motion as an attribute and the challenge of binding a common element in a succession of incoming signals, a more elementary analysis would initially focus on the one-time display of a single external object. The aim here is to identify instantiations, *in concreto*, as Kant liked to say, of entities in his system where a unified representation of this object has emerged. His use of the term *Vorstellung* is too wide to serve as a guide for this restricted meaning.

The raw sensory impressions, lacking structure, obviously do not qualify, but *manifolds*, the results of space and time formatting and productive imagination synthesis, do. Kant is very insistent of their unity in appearance and, they being *Anschauungen*, does not deny them a presence in consciousness.

Forward transfer from the level of sensibility to that of understanding and simultaneously operated on by *Schemata* mediating the influence of the *Categories*, a new entity is synthesized, which differs fundamentally from *Manifolds* by being understood, comprehended, cognized; it becomes an *Object in Cognition*, i.e. an object that has been comprehended.

Kant goes yet a little further, by differentiating between a perceived object as a single understood object, and a binding with other concepts that in addition provide a relation to the experiencing subject, i.e. he takes the step from perception to *apperception*.

In summary, three distinct representations of an external object seem sufficiently sharply delineated in the *Critique* that they can be proposed as instantiable constructs for purposes of material/mental correlation: *manifolds in the Anschauung, comprehended objects* in the realm of Verstand, and those which have additionally been related to the experiencer, i.e. have been *apperceived*.

To provide a graphic illustration, we can go to Kant's dog example (*B180*). In the external world before me is my dog, *Rover*. The *manifold* has the “*Gestalt of a four-footed animal*,” the *object in cognition* is a *dog*, the *apperception* is that of *my dog, Rover.*

## Discussion

Although much philosophy is devoted to the existence of a real world, Kant magisterially brushes this question aside, in favor of positing two realms separated by a barrier, the mental world being immediate to us as individuals, the material world only via sensory impressions. He leaves it to the natural scientists to build models of the material world and devotes his efforts to develop a comprehensive, textured and nuanced system of constructs and processes in the mental world.

Limitations must be admitted when viewing 250-year old propositions through a modern lens. A specific problem is consciousness, much argued over, even by Kant himself. But as has been evident, going back to at least Freud, what is mental does not necessarily have to be conscious. Hence, “experimental phenomenology,” insofar as it defined as the correlation of brain states with the content of consciousness, is not synonymous with cognitive neuroscience. Entirely missing in Kant's system are also two aspects on which much modern research is concentrated, attention, and plasticity. He occasionally addresses the time dimension but largely in connection with the persistence needed to unify and firm up his sensory constructs; these, for purposes of the present discussion, would be regarded as stationary.

Kant's relevance to present-day neuroscientists lies in laying out a full structure of the mental world. For example, *Schemata*, a synthesizing process of an empirically apprehended object in perception with previously existing elements cannot but describe the recognition process associated with object perception. Another is *creative imagination,* a top-down influencing stream leading to synthesis of incoming sensory signals by pre-existing rules. This prescience of Gestalt theory is remarkable. In 1920 the proposition was explicitly made by Köhler that his phenomenal laws of Gestalt might find corresponding “brain observations … by physical means”:

*“In principle, a brain observation is conceivable, which would recognize similarities in Gestalt- and therefore in the most essential properties by physical means with what the subject experiences phenomenally.*” (Köhler, 1920 p. 193).

Köhler's own attempts, 100 years ago, were premature, but the situation has vastly changed through the giant strides made in experimental techniques of recording cortical neural activity by non-invasive means in the alert subject. To effectively pursue these lines of investigation, insight is also needed into the “essential properties [of] .., what the subject experiences phenomenally.” This is territory not easily accessible to those trained in classical neurophysiology and is still in need of delineation matching in rigor those more commonly employed there. Fechnerian psychophysics fulfills this requirement but much in, say, Gestalt theory, does not. The task is now to endow proposed mental processes and conjectured structures with the kind of properties that fits them into experimental protocols and thus promises for them the kind of solidity that is the ideal in natural science. The first step in this direction must be the identification of candidate elements, entities, or streams that might satisfy the criteria of being sufficiently sharply definable and suitable for the neural/mental correspondence envisaged by Köhler and included in the mission of cognitive neuroscience. Building on Kant's system would seem to be a promising start. Actually Kant's landscape is much more encompassing than the area here examined. But perception and cognition of visual objects are major constituents of it, as well as topics that have a long tradition of analysis in both the physical and mental worlds.

## Conclusion

Firmly insisting on a separation of mental and material worlds, Kant cedes analysis of the material world to natural scientists and develops a model of the mental world with hierarchical domains and streams of interaction between constructs. An abiding hope of neuroscientists has been to find correlation between physically defined brain states and phenomenally experienced mental states. Enormous methodological advances in neuroscience are now poised for empirical research in this area. Kant's taxonomy of mental entities and streams, not easily accessible in his writings, is here sketched in a more contemporary idiom to enable the defining of sufficiently delineated mental states to serve as targets for correlation.

## Glossary

*Anschauung*: B33/376: elements immediately given in the realm of sensibility
*Apperzeption B132f*: Apperception conscious perception related to oneself
*Begriff*: B376: concept
*Einbildungskraft B152*: productive imagination synthetic influence of Verstand on inner sense
*Empfindung*: B376: sensation: modification of subject's state
*Erkenntnis*: B376: cognition, knowledge: objektive Perzeption
*Erscheinung*: B34: appearance
*Idee*: B376: idea: concept in the realm of reason
*Mannigfaltige*: B105: manifold
*Perception*: B376: perception: conscious Vorstellung
*Sinneseindruecke*: sensory impressions
*Sinnlichkeit*: B33: sensibility
*Vernunft*: reason
*Verstand*: understanding
*Vorstellung*: representation
